# A Pragmatic Study Shows Failure of Dental Composite Fillings Is Genetically Determined: A Contribution to the Discussion on Dental Amalgams

**DOI:** 10.3389/fmed.2017.00186

**Published:** 2017-11-06

**Authors:** Alexandre R. Vieira, Marília B. Silva, Kesia K. A. Souza, Arnôldo V. A. Filho, Aronita Rosenblatt, Adriana Modesto

**Affiliations:** ^1^Departments of Oral Biology, University of Pittsburgh School of Dental Medicine, Pittsburgh, PA, United States; ^2^Departments of Pediatric Dentistry, University of Pittsburgh School of Dental Medicine, Pittsburgh, PA, United States; ^3^Department of Preventive Dentistry, University of Pernambuco School of Dentistry, Recife, Pernambuco, Brazil

**Keywords:** dental caries, matrix metalloproteinases, dental amalgam, composite resin, linkage disequilibrium

## Abstract

Composite resins for posterior tooth restorations have become a viable alternative to dental amalgam. Failures sometimes cannot be easily explained, and we hypothesize that a genetic component may influence longevity of restorations. We aimed to determine if there is any evidence for a difference in the performance of amalgams versus composite resin in extensive posterior restorations. We also aimed to determine if risk factors such as age, sex, smoking tobacco, alcohol drinking, diabetes status, and periodontal health status may have a role in the failures of extensive anterior composite restorations. Finally, we investigated if genetic variation in matrix metalloproteinases that are present in the mineralized dentin is associated with failure of composite resin. The data used to perform this research were obtained from the Dental Registry and DNA Repository project after screening 4,856 patients. All restorations were evaluated at times of 1, 2, and 5 years after the restoration placement. 6,266 amalgam and 2,010 composite restorations were analyzed in a total of 807 patients in a period of approximately 10 years (period corresponding to the database existence). An additional 443 extensive direct composite resin restorations in anterior teeth were also studied. Failure rates of amalgam and composite restorations are similar, and by the end of 5 years, composites outperformed amalgams slightly. Failures of anterior composite restorations occurred more often in males who smoked tobacco (*p* = 0.05), despite a similar number of females and males that smoked tobacco in the sample (116 individuals smoked tobacco, 54 females and 62 males). Alcohol drinking increased failure rate within 2 years (*p* = 0.03). We found a statistically significant association between matrix metalloproteinase 2 rs9923304 and failure of composite restorations (*p* = 0.007). Composite resins can replace amalgam restorations. Smoking tobacco and drinking alcohol will increase the chance of restoration failure.

## Introduction

In 2008, Norway was the first country to completely ban amalgam dental restorations, quickly followed by Sweden and Denmark. This decision was motivated by concerns related to the composition of the amalgam and the existence of viable non-mercury filling substitutes (composite resins for the most part). Currently, amalgam still has widespread use. Concerns regarding amalgam substitutes are related to their longevity in the mouth (1–4).

Nevertheless, dentists appear to favor using amalgam in more challenging cases (5). No empirical evidence exists that supports the assumption that dental amalgam restorations can lead to neurotoxic and nephrotoxic effects in humans (6). When dental disease is taken into consideration, large composite restorations survive longer in individuals with low risk for disease in comparison with individuals with concomitant diseases. For high risk patients, amalgam shows better survival (7). Despite the evidence showing no health consequences for having dental amalgam restorations, the question persists regarding composites being able to provide the same performance of their metallic counterparts. Since we have a registry of dental clinical information linked to biological samples, we aimed to determine if there is any evidence for a difference in the performance of amalgams versus composite resin in extensive posterior restorations. We also aimed to determine if risk factors, such as age, sex, smoking tobacco, alcohol drinking, diabetes status, and periodontal health status, may have a role in the failures of extensive anterior composite restorations. Finally, we investigated if genetic variation in matrix metalloproteinases (MMPs) that are present in the mineralized dentin is associated with failure of composite resin. In this study, we show that composite resins can fully substitute dental amalgams in routine dental practice with the likely benefit of not having any environmental consequences. In case of anterior complex direct composite resin restorations, we show that smoking tobacco and drinking alcohol will increase the chance of restoration failure. Finally, since dentin MMPs are exposed and activated during the process of restoring a tooth with composite resin, they degrade type I collagen (8–11), we tested if genetic variation in MMPs are associated with composite restoration failures and show that the matrix metalloproteinase 2 (*MMP2*) may have a role in the failure of composite restorations.

## Materials and Methods

The data studied here were obtained from the University of Pittsburgh School of Dental Medicine Dental Registry and DNA Repository project. The primary role of the School of Dental Medicine is to educate dental health professionals and students depending on the patient’s commitment to complete necessary educational requirements. These patients are the ones that are invited to participate in the Dental Registry and DNA Repository project. Patients treated are, for the most part, from the great Pittsburgh area but the patient pool tends to be overrepresented by individuals with lower social economic status and hence higher risk for all oral and overall health issues. However, the population treated at the School of Dental Medicine and the individuals participating in the registry are a better representation of the ethnic distribution of individuals of Pittsburgh, which is comprised by approximately 65% Whites, 26% African Americans and the remaining a mix of Hispanics, Asians, and other groups. Starting in September of 2006, all individuals who sought treatment at the University of Pittsburgh School of Dental Medicine were invited to be part of the registry. These individuals gave written informed consent authorizing the extraction of information from their dental records and collection of a saliva sample. Saliva samples were stored for future studies and served as source of DNA. This project was approved by the University of Pittsburgh Institutional Review Board (IRB # 0606091). This manuscript follows the STROBE guidelines for reporting observational studies.

**Figure 1:**
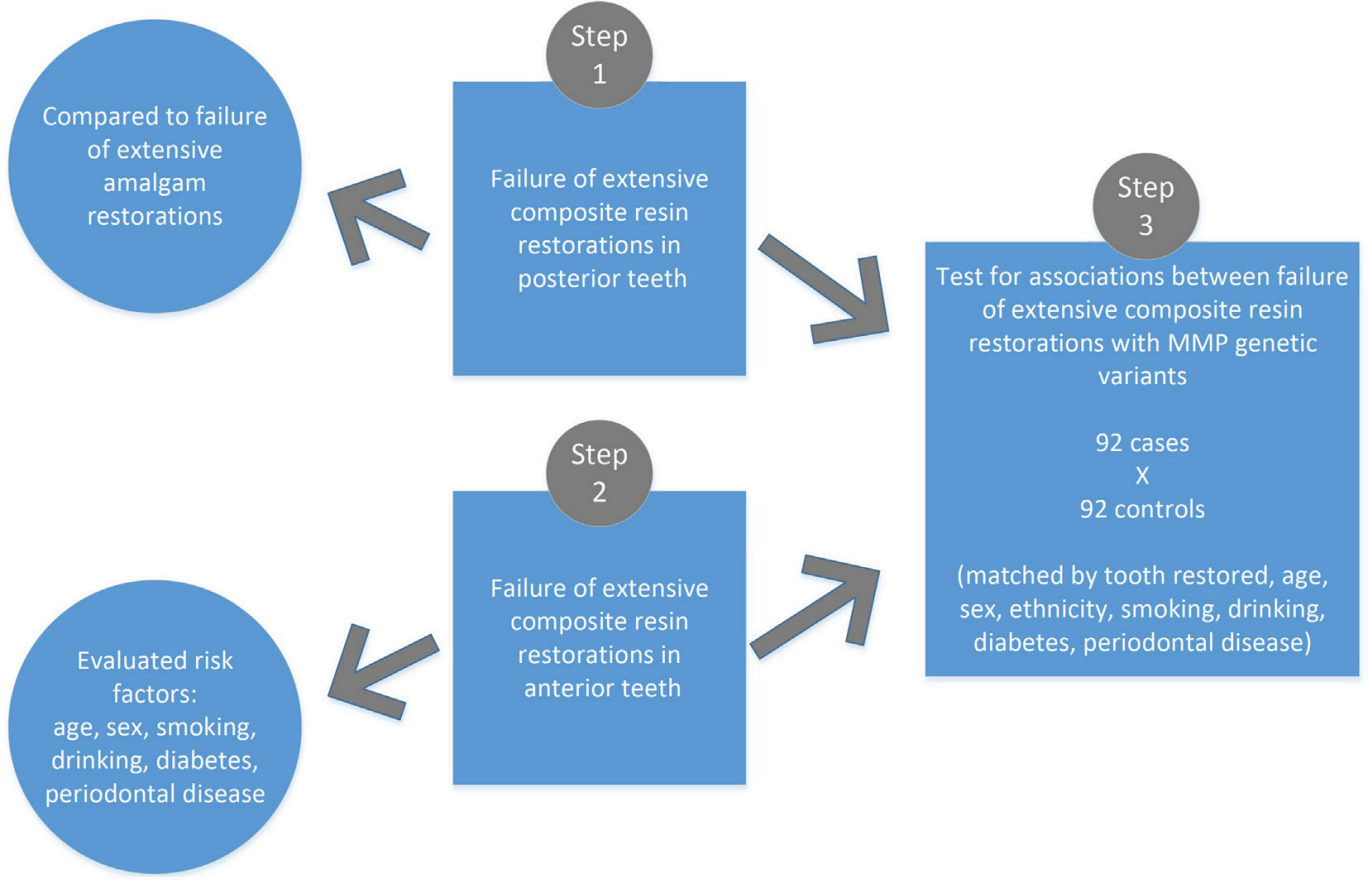
Summary of the study design.

**Figure 2:**
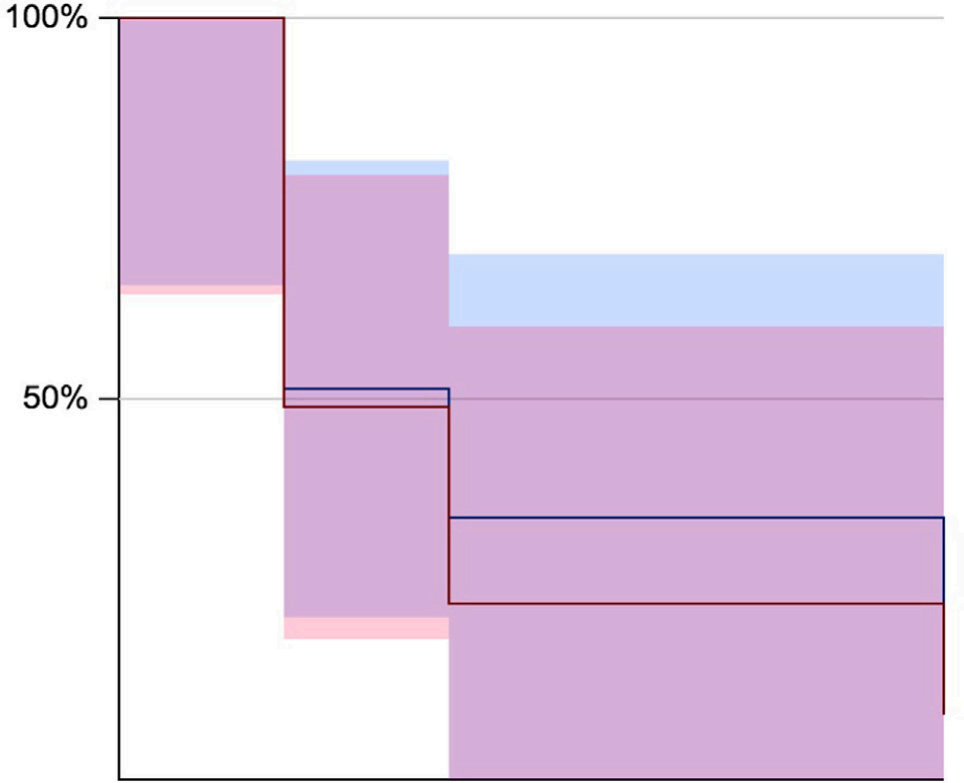
Kaplan–Meier survival estimates for 1, 2, and 5 years (blue for amalgam restorations and in pink composite resin restorations). Corresponding data can be found in Table 2.

For this study, 4,856 patient records that are part of our registry were screened. Figure 1 provides an overall picture of our study. In the first step, all restorations of posterior teeth involving the occlusal and at least one proximal surface were evaluated and their status was determined at 1, 2, and 5 years after placement of the restorations. Data extracted included date of placement and date of the observed failure (as determined by a reevaluation of the case). Restorations lost at follow-up were not included in the analysis [2,054 (32.78%) amalgam and 781 (38.85%) composite restorations]. 6,266 amalgam and 2,010 direct composite resin restorations were analyzed in a total of 807 patients (434 females, 373 males, mean age 42 years) in a period of approximately 10 years (period corresponding to the database existence) (Table 1). Restoration failure was defined as necessity of replacement due to fracture, discoloration in the case of composites, or development of secondary caries. The goal of this comparison was to determine if there are any differences in the rates of failure of these two types of restorations. Aside from standard non-parametric analysis (i.e., chi-square), we also performed a Kaplan–Meier survival probability estimate (12–14) using the VassarStats package (15) (Table 2, Figure 2). It was not possible to precise the month in which the restoration failed for all cases since patients not necessarily visited the dentist right away. Therefore, we decided to group the observations by three time ranges as described above.

**Table 1:** Age, sex, and caries experience of the study population.

Posterior tooth type and material used to restore	Sex	Age range in years	Number of subjects	Mean age in years	Caries experience (mean DMFT score)
Molars restored with amalgam	Females	20–30	159	25.99	13.8
		31–40	146	34.69	14.12
		41–50	101	46.54	16.13
		51–60	154	56.03	16.87
		Total	560	40.81	15.23

	Males	20–30	129	26.16	14.85
		31–40	119	34.39	14.23
		41–50	85	45.1	15.2
		51–60	153	55.18	17.37
		Total	486	40.21	15.41

Molars restored with direct composite resin	Females	20–30	101	25.95	10.57
		31–40	59	33.3	13.02
		41–50	28	46.28	15.57
		51–60	57	55	15.24
		Total	245	40.13	13.6

	Males	20–30	57	26.38	11.16
		31–40	29	34.34	15.34
		41–50	16	45.94	15.56
		51–60	30	55.97	15.4
		Total	132	40.66	14.36

Premolars restored with amalgam	Females	20–30	131	25.92	14.54
		31–40	117	34.42	16
		41–50	84	46.33	17.38
		51–60	137	55.62	18.19
		Total	469	40.57	16.53

	Males	20–30	117	26.01	15.12
		31–40	99	34.13	16.02
		41–50	66	45.59	17.03
		51–60	135	55.65	18.95
		Total	417	40.34	16.78

Premolars restored with direct composite resin	Females	20–30	102	26.26	15.12
		31–40	84	33.51	16.02
		41–50	32	46.03	17.03
		51–60	60	55.75	18.95
		Total	278	40.39	16.78

	Males	20–30	65	25.74	13.95
		31–40	54	33.96	15.7
		41–50	30	45.37	16.17
		51–60	47	55.89	17.89
		Total	196	47.74	15.93

*DMFT, decayed, missing due to caries, filled teeth*.

**Table 2:** Kaplan–Meier survival probabilities estimates.

Timespan	Restorations at risk	Restorations that failed	Probability	95% Confidence interval
**Amalgam**				
Up to 1 year	4,212	612	0.85	0.84–0.86
1–2 years	3,600	134	0.82	0.81–0.83
2–5 years	3,466	203	0.77	0.76–0.79
**Composite resin**				
Up to 1 year	1,229	198	0.83	0.82–0.86
1–2 years	1,031	58	0.79	0.77–0.81
2–5 years	973	24	0.77	0.74–0.79
Log-rank test			*Z* = 0.75	*p* = 0.45

In the second step, we selected patients that have at least one complex restoration of anterior teeth that involves four or more surfaces. From the registry records available, 412 patient records included an extensive anterior direct composite resin (210 females, mean age of 44.95 years, and 202 males, mean age of 46.37 years); however, we excluded 139 cases in which the restoration was not performed in our clinics and hence no baseline could be determined. A total of 443 direct composite restorations of anterior teeth were studied. Failed restorations were defined as described above. The goal of this second analysis was determining if risk factors, such as age, sex, smoking tobacco (116 individuals smoked tobacco, 54 females and 62 males), alcohol drinking (28 individuals drank alcohol, 7 females and 21 males), diabetes status (34 individuals were diabetic, 11 females and 23 males), and periodontal health status (252 had a diagnosis of periodontitis, 112 females and 140 males) played a role in direct composite restorations of anterior teeth failures. We used chi-square and calculated odds ratios (ORs) and 95% confidence intervals (CIs) comparing individuals who carried a certain risk factor compared to individuals without the risk factor.

In the last step, we studied the cases in which composite resin restorations failed; a total of 92 unrelated individuals who had a failed composite resin (either in the anterior or posterior teeth). For this part of the study, we tested the hypothesis that genetic variation in specific MMP genes is overrepresented in cases of failed composite resin restorations. For comparison, we selected from our registry 92 unrelated individuals (50 females, 42 males, mean age 41 years) matched by type of composite restoration (same tooth, same dental surfaces involved), age, sex, ethnicity, smoking tobacco, drinking alcohol, diabetes, and periodontal health. The rationale for this last experiment is that MMPs are exposed and activated by the acidic agents during adhesive bonding procedures prior to placement of direct composite resin restorations. Our overall hypothesis is that carrying specific MMP alleles increases degradation of the collagen fibrils at the resin–dentin-bonded interfaces, which leads to clinical failure of composite restorations.

We studied 20 single nucleotide polymorphisms in four genes (Table 3): *MMP2* (rs9923304, rs2285053, rs243865, rs2287074, rs243847, rs11639960, rs2241145, rs243832), *MMP3* (rs650108, rs520540, rs639752, rs679620, rs522616, rs520540), *MMP8* (rs3765620, rs17099443), and *MMP9* (rs17576, rs2272766, rs13925, rs2236416). These genetic variants were selected due to their known functional activity, gene location, and allele frequency (16–19). Genotyping data for the 92 individuals selected as cases and 92 individuals used as comparison were commensurate with genotyping data obtained previously from distinct individuals from the Pittsburgh area drawn for studies of different phenotypes [formation of periapical lesion related to deep caries lesions in dentin (16); chronic periodontitis (18)] and we concluded these samples we selected were not under the influence of undetected population substructure.

**Table 3:** Genetic markers studied.

Gene and gene function	Genetic marker	Location in the gene	Summary result of association (*p*-value)
Matrix metalloproteinase 2 (*MMP2*) gelatinase A, type IV collagenase	rs9923304	Intronic	0.007
	rs2285053	Flanking 5′ end	0.13
	rs243865	Flanking 5′ end	0.52
	rs2287074	Exon, silent mutation	0.01
	rs243847	Intronic	0.05
	rs11639960	Intronic	0.62
	rs2241145	Intronic	0.01
	rs243832	Intronic	0.87
*MMP3* stromelysin 1	rs650108	Intronic	0.75
	rs520540	Exon, silent mutation	0.3
	rs639752	Intronic	0.06
	rs679620	Exon, missense mutation	0.57
	rs522616	Flanking 5′ end	0.99
	rs520540	Exon, silent mutation	0.93
*MMP8* neutrophil collagenase	rs3765620	Exon, silent mutation	0.55
	rs17099443	Intronic	0.45
*MMP9* gelatinase B, type IV collagenase	rs17576	Exon, missense mutation	0.49
	rs2272766	Intronic	0.64
	rs13925	Exon, silent mutation	1.0
	rs2236416	Intronic	0.85

*Information about the markers studied can also be found at*:

*MMP2: http://www.gwascentral.org/generegion/markers?q=mmp2&t=ZERO&fpf=0&tpf=0&l=asd&r%5B%5D= or http://identifiers.org/dbsnp/rs9923304; http://identifiers.org/dbsnp/rs2285053; http://identifiers.org/dbsnp/rs243865; http://identifiers.org/dbsnp/rs2287074; http://identifiers.org/dbsnp/rs243847; http://identifiers.org/dbsnp/rs11639960; http://identifiers.org/dbsnp/rs2241145; http://identifiers.org/dbsnp/rs243832*.

*MMP3: http://www.gwascentral.org/generegion/markers?q=mmp3&t=ZERO&fpf=0&tpf=0&l=asd&r%5B%5D=&page=1&o=1&page_size=50&format= or http://identifiers.org/dbsnp/rs650108; http://identifiers.org/dbsnp/rs520540; http://identifiers.org/dbsnp/rs639752; http://identifiers.org/dbsnp/rs679620; http://identifiers.org/dbsnp/rs522616; http://identifiers.org/dbsnp/rs520540*.

*MMP8: http://www.gwascentral.org/generegion/markers?q=mmp8&t=ZERO&fpf=0&tpf=0&l=asd&r%5B%5D=&page=1&o=1&page_size=50&format= or http://identifiers.org/dbsnp/rs3765620; http://identifiers.org/dbsnp/rs17099443*.

*MMP9: http://www.gwascentral.org/generegion/markers?q=mmp9&t=ZERO&fpf=0&tpf=0&l=asd&r%5B%5D=&page=1&o=1&page_size=50&format= or http://identifiers.org/dbsnp/rs17576; http://identifiers.org/dbsnp/rs2272766; http://identifiers.org/dbsnp/rs13925; http://identifiers.org/dbsnp/rs2236416*.

Chi-square and Fisher’s exact tests, and OR with 95% CI calculations were used to determine Hardy–Weinberg equilibrium and statistically significant differences with an alpha of 0.05. The PLINK software package (20) was used to analyze the distribution of alleles and genotypes between individuals with direct composite resins that failed versus controls that had the same restorations without any failures. To assure, we obtained a satisfactory matching for our case–control analysis, we used age, sex, ethnicity, smoking tobacco, drinking alcohol, diabetes, and periodontal health as covariates and performed both linear and logistic analyses and found that there were no differential influence of the covariates in the results. Therefore, we present here the results of the genotypic (two degrees of freedom) test as implemented in PLINK.

## Results

### Amalgam versus Composite Resin

From those 6,266 amalgam and 2,010 composite restorations, 2,054 amalgam and 781 composite restorations could not be monitored, since the patients discontinued treatment. A total of 612 amalgam (9.76%) and 198 composite (9.85%) restorations were considered failures in the period up to 1 year after the placement of the restoration (*p* = 0.08), 134 amalgam (2.14%) and 58 composite (2.88%) restorations between 1 and 2 years (*p* = 0.38), and 203 amalgam (3.24%) and 24 composite (1.19%) restorations within 2–5 years (*p* = 0.0000001). These figures show lower rates than those found in the literature (4) and show small differences between up to 5-year failure rates of amalgam in comparison to composite restorations. The differences in failure rates of 0.09% in favor of amalgam up to 1 year of follow-up, 0.74% in favor of amalgam up to 2 years of follow-up, and 2.05% in favor of direct composite resins up to 5 years of follow-up appear to be relatively small differences of one material over the other; however, the impact of a couple percent points may be substantial when costs are accounted for large population numbers. Kaplan–Meier survival probabilities estimates showed no differences in the survival of amalgam versus composite resin for extensive restorations of posterior teeth (Table 2; Figure 2), and were done just to illustrate the initial findings. Since we have grouped subjects in three representative timeframes (1, 2, and 5 years), these data do not show survival by shorter intervals. It is worth noting that these restorations were performed by students (dentists in training). The less technique sensitive dental amalgam performance was similar to the technique sensitive composite resin in our student’s hands, which contradicts previous reports suggesting composite resins are 10 times more likely to fail when placed by dental students (21).

### Anterior Complex Composite Resin Restorations

A total of 253 individuals who had 443 direct composite resin restorations were studied. Among these individuals, males were more commonly affected by diabetes (*p* = 0.006) and periodontal disease (*p* = 0.006) than females. From the total number of anterior complex restorations, 41 were considered failure (9%). Conversely, 268 restorations were considered satisfactory over a period of 6 months to 2 years, 78 during the period from 2 to 4 years, and 56 over 4 years. The main reasons for restoration failure were secondary caries (*N* = 19), extraction due to reasons other than caries or fracture (*N* = 11), esthetic issues (*N* = 7), and fracture (*N* = 4). Failures occurred more often in males who smoked tobacco [*p* = 0.05; OR = 1.15 (95% CI 1.0–1.4)]. Failures were five times more common in the maxilla in patients who did not smoke tobacco; however, smoking tobacco changed the maxilla–mandible ratio to 1:1. Finally, alcohol drinking increased failure rate within 2 years [*p* = 0.03; OR = 4.4 (95% CI 1.2–16.1)].

### Genetic Association between MMPs and Direct Composite Resin Restoration Failures

All genotypes were in Hardy–Weinberg equilibrium. The case–control analyses showed a statistically significant association between *MMP2* rs9923304 and failure of composite restorations (*p* = 0.007). The *MMP2* rs2287074 (*p* = 0.01), rs243847 (*p* = 0.05), and rs2241145 (*p* = 0.01) variants also showed association with failure of composite restorations. One variant in *MMP3* (rs639752) had a *p*-value borderline of the nominal alpha of 0.05 (*p* = 0.06). The distribution of all other genetic variants was not statistically and significantly different.

## Discussion

Mineralized dentin contains MMP2, MMP3, MMP8, and MMP9 and acidic resin components incorporated into etch-and-rinse adhesives and self-etch adhesives increase collagenolytic and gelatinolytic activities of demineralized collagen matrices. Apparently, MMP2 and MMP9 are involved in the degradation process [reviewed by Liu et al. (22)]. We show for the first time that genetic variation potentially affecting degradation of collagen is associated with failures of more extensive composite restorations. *MMP2* rs9923304 TT genotype frequency is 23% in Whites, 14% in Chinese and Japanese, and 1.8% in Yoruba Africans (according to the dbSNP: http://www.ncbi.nlm.nih.gov/projects/SNP/snp_ref.cgi?rs=9923304) and could serve as the basis for a genomic approach to define risks for shorter longevity of extensive composite restorations and more frequent recalls of patients treated with direct composite resins. These results support the suggestion that patient-related factors rather than the restorative material are the most important determinants of secondary caries and restoration failures (23). It is remarkable that the association we found between *MMP2* rs9923304 and failure of extensive composite restorations is evident with only 92 cases. While concerned about multiple testing, we did not apply the strict Bonferroni correction as it would increase type II errors and a major focus of this study was to identify putative associations with having a failure composite resin for further studies. For example, under the Bonferroni correction, we would have lowered the alpha to 0.0025 (0.05/20) and the result of rs9923304 (*p* = 0.007) would be considered not significant. We have demonstrated before (24) that known true associations are missed when correction for multiple testing is implemented. It is also important to note that a larger discussion on the interpretation of *p*-values is currently underway and we should move to the near future to an interpretation that takes into account the biological relevance of an observation beyond the blind consideration of the arbitrary alpha threshold to determine significance (25). The results of our work should be considered with caution and serve to generate hypothesis to be directly tested in larger and more homogeneous samples. On the other hand, simply disregarding the nominal associations presented here may delay discovery by misleading the field to believe no true biological relationships exist. Whereas the *p*-value of 0.007 may not reach the strict Bonferroni threshold that would be applied to our analysis, the presence of four genetic variants in *MMP2* with nominal associations at a 0.05 alpha level suggest a role for this gene in dentine degradation and composite resin failure. We also simplified our analyses (bivariate instead of multivariate) since we paired our sample by a number of covariates and each individual included was not related to anyone else in the cohort.

Our data also show that direct composite resins perform similarly (and maybe slightly better) to amalgam in posterior teeth up to 5 years and are suitable substitutes for their metallic counterparts, making it feasible to completely replace amalgam in dentistry. The justification of using amalgam due to its lower costs alone in contrast to the potential of eliminating an environmental hazard has become harder to support now that direct composite resins can perform at acceptable levels. Our data come from a large clinic where dentists working are in the beginning of their professional careers. Also, the population treated has some of the worst oral health and overall health indicators in the country (26). The statistically significant lower failure rate of posterior composite resin versus amalgam restorations with 5 years follow-up in our study, despite the fact that direct composite resins are more technique sensitive than amalgam, further suggest that the first can replace the latter. We also showed that smoking tobacco and alcohol drinking increase the chance of anterior extensive direct composite resin restoration failure. Based on these data, we argue that the mechanism underlying this result relates to the fact that smoking tobacco alters salivation (27), since we found that the higher failure rates of anterior maxillary restorations in the cases when individuals did not smoke equalized with anterior mandibular restoration failures when the patient smoked tobacco. Earlier reports from the literature do not suggest smoking negatively impact the clinical performance of resin composite cervical restorations up to 1 year (28).

Matrix metalloproteinase 2, also known as gelatinase A, is a membrane-bound protein that is important for extracellular matrix turnover, preferentially cleaving collagen types IV, V, VII, and XI and gelatin (29). The *MMP2* variant rs9923304 was found to be associated with formation of a periapical lesion when there is deep carious lesions in dentin (16). The underlying mechanism for this finding is possibly the same that explains the association of this genetic variant in *MMP2* and failures of direct resin composite restorations of the present study. MMP2 gelatinolytic activity probably occurs both in partially demineralized dentin at the bottom of caries lesions and at the surface treated with either etch-and-rinse or self-etch adhesives. Overtime, this gelatinolytic activity weakens the bond between the material and the remaining dentin structure leading to failure of the restoration. This result is compatible with the findings that showed significantly higher risk of failure of composite restorations in individuals with a higher number of restored surfaces (3).

Altered expression and activity levels of MMP2 is known to be associated with pathological states. For instance, in colorectal cancer, *MMP2* mRNA is detected in higher levels in unaffected tissue surrounding metastatic tumors (30). Inactivating mutations in *MMP2* in humans lead to connective tissue underlying issues as seem in Torg–Winchester syndrome (31), multicentric osteolysis, arthritis syndrome (32), and possibly influence the formation of keloids (33). *MMP2* encodes an enzyme that degrades type IV collagen. This role is particularly important in endometrial menstrual breakdown, vascular regulation, and inflammatory responses (34). In teeth, type IV collagen is located along the dental–enamel junction (35). Therefore, knowing the roles of MMP2 in other tissues and pathologies, we believe the mechanism underlying the genetic influence of *MMP2* in failure of extensive composite resin restorations involves degradation of type IV collagen in individuals who have higher *MMP2* activity for carrying particular genetic variants in the gene, which is located along of the dentin–enamel junction. Essentially, this would undermine the whole extension of the restoration right below its enamel margins, at the portions of dentin closest to enamel, a band approximately 5- to 10-µm wide (35). Although *MMP2* rs9923304 may not have any function, it may be a surrogate for individuals at higher risk for extensive composite resin restorations failure since it may be in linkage disequilibrium to genetic variants that alter *MMP2* activity.

Since MMP activity has been associated with severity of chronic airway diseases (36), it would be interesting to investigate if patients with conditions such as idiopathic interstitial pneumonia or bronchiectasis have a history of more often replacing direct composite resins or forming periapical lesions in comparison to individuals not affected by chronic airway diseases. Similarly, since MMP2 activity has been linked to poor prognosis of multiple forms of cancer (37), including colorectal, melanoma, breast, lung, ovarian, and prostate, it would be interesting to interrogate if patients that more often have direct composite resin failures or develop periapical lesions, when affected by cancer, have a worse prognosis.

In summary, our studies of cases with extensive direct composite resin restoration failures suggest that composite resins can fully substitute dental amalgams in routine dental practice. Smoking tobacco and alcohol drinking increase the chance of failure in anterior complex direct composite resin restorations. Finally, *MMP2* may have a role in the cases that composite restorations fail and genotyping rs9923304 may be useful to determine follow-ups of extensive direct composite resin restorations.

## Ethics Statement

This study was carried out in accordance with the recommendations of the Belmont Report, University of Pittsburgh institutional Review Board with written informed consent from all subjects. All subjects gave written informed consent in accordance with the Declaration of Helsinki. The protocol was approved by the University of Pittsburgh Institutional Review Board.

## Author Contributions

AV designed the studies, obtained support, analyzed and interpreted data, and wrote the first draft of the manuscript. MS, KS, and AF generated data, analyzed and interpreted data, and critically revised the manuscript. AR and AM obtained support, interpreted data, and critically revised the manuscript.

## Conflict of Interest Statement

The authors declare that the research was conducted in the absence of any commercial or financial relationships that could be construed as a potential conflict of interest.
